# Whole exome and genome sequencing in mendelian disorders: a diagnostic and health economic analysis

**DOI:** 10.1038/s41431-022-01162-2

**Published:** 2022-08-15

**Authors:** Lisa J. Ewans, Andre E. Minoche, Deborah Schofield, Rupendra Shrestha, Clare Puttick, Ying Zhu, Alexander Drew, Velimir Gayevskiy, George Elakis, Corrina Walsh, Lesley C. Adès, Alison Colley, Carolyn Ellaway, Carey-Anne Evans, Mary-Louise Freckmann, Linda Goodwin, Anna Hackett, Benjamin Kamien, Edwin P. Kirk, Michelle Lipke, David Mowat, Elizabeth Palmer, Sulekha Rajagopalan, Anne Ronan, Rani Sachdev, William Stevenson, Anne Turner, Meredith Wilson, Lisa Worgan, Marie-Christine Morel-Kopp, Michael Field, Michael F. Buckley, Mark J. Cowley, Marcel E. Dinger, Tony Roscioli

**Affiliations:** 1 St Vincent’s Clinical School, University of New South Wales, Sydney, NSW Australia; 2 Kinghorn Centre for Clinical Genomics, Garvan Institute of Medical Research, Sydney, NSW Australia; 3 Centre for Clinical Genetics, Sydney Children’s Hospital, Sydney, NSW Australia; 4 Centre for Economic Impacts of Genomic Medicine, Macquarie Business School, Macquarie University, Sydney, NSW Australia; 5 The Genetics of Learning Disability Service, Newcastle, NSW Australia; 6 Randwick Genomics Laboratory, NSW Health Pathology, Prince of Wales Hospital, Sydney, NSW Australia; 7 Department of Clinical Genetics, Children’s Hospital at Westmead, Sydney, NSW Australia; 8 Disciplines of Child and Adolescent Health and Genomic Medicine, University of Sydney, Sydney, NSW Australia; 9 Clinical Genetics Department, Liverpool Hospital, Sydney, NSW Australia; 10 Neuroscience Research Australia (NeuRA) and Prince of Wales Clinical School, UNSW, Sydney, Australia; 11 Clinical Genetics, Royal North Shore Hospital, Sydney, NSW Australia; 12 Genetics Services, Nepean Hospital, Sydney, NSW Australia; 13 Hunter Genetics, Newcastle, NSW Australia; 14 School of Women’s and Children’s Health, University of New South Wales, Sydney, Australia; 15 Queensland Children’s Hospital, Brisbane, QLD Australia; 16 Northern Blood Research Centre, Kolling Institute of Medical Research, University of Sydney, Sydney, NSW Australia; 17 Department of Haematology and Transfusion Medicine, Royal North Shore Hospital, Sydney, NSW Australia; 18 School of Biotechnology and Biomolecular Sciences, University of New South Wales, Sydney, Australia

**Keywords:** Genetics research, Genetic testing, Medical genomics

## Abstract

Whole genome sequencing (WGS) improves Mendelian disorder diagnosis over whole exome sequencing (WES); however, additional diagnostic yields and costs remain undefined. We investigated differences between diagnostic and cost outcomes of WGS and WES in a cohort with suspected Mendelian disorders. WGS was performed in 38 WES-negative families derived from a 64 family Mendelian cohort that previously underwent WES. For new WGS diagnoses, contemporary WES reanalysis determined whether variants were diagnosable by original WES or unique to WGS. Diagnostic rates were estimated for WES and WGS to simulate outcomes if both had been applied to the 64 families. Diagnostic costs were calculated for various genomic testing scenarios. WGS diagnosed 34% (13/38) of WES-negative families. However, contemporary WES reanalysis on average 2 years later would have diagnosed 18% (7/38 families) resulting in a WGS-specific diagnostic yield of 19% (6/31 remaining families). In WES-negative families, the incremental cost per additional diagnosis using WGS following WES reanalysis was AU$36,710 (£19,407;US$23,727) and WGS alone was AU$41,916 (£22,159;US$27,093) compared to WES-reanalysis. When we simulated the use of WGS alone as an initial genomic test, the incremental cost for each additional diagnosis was AU$29,708 (£15,705;US$19,201) whereas contemporary WES followed by WGS was AU$36,710 (£19,407;US$23,727) compared to contemporary WES. Our findings confirm that WGS is the optimal genomic test choice for maximal diagnosis in Mendelian disorders. However, accepting a small reduction in diagnostic yield, WES with subsequent reanalysis confers the lowest costs. Whether WES or WGS is utilised will depend on clinical scenario and local resourcing and availability.

## Introduction

Genomic technologies have improved Mendelian disorder diagnosis, with whole genome sequencing (WGS) having the greatest diagnostic yield [1–3]. The higher cost of WGS sequencing and long-term data storage remain barriers to its routine implementation. Without public funding for genomic testing in most countries, diagnostic yields are balanced against budgetary limitations. The impact of coding variation on gene function identified through whole exome sequencing (WES) and WGS is well understood. The advantages of WGS for improving diagnostic yield are coding region coverage consistency, sequencing of newly-annotated coding regions, and improved detection sensitivity for structural variants (SVs), particularly copy number variants (CNVs) [2]. Interpreting genetic variation in non-coding regions identified primarily through WGS remains challenging, leading to a perceived lack of additional WGS utility compared to WES [4], however several reports have identified non-coding causes of Mendelian diseases [5–7].

While WGS increases the diagnostic yield over WES in Mendelian disorders, there are few studies exploring the degree of improvement. Such studies would assist in selecting the optimal clinical genomic investigation. A small number of studies have assessed WGS diagnostic yields in WES-negative Mendelian disorder cohorts, with diagnostic rates between 7 to 34% [8–11]. The increased diagnostic rate in these studies was due to CNV detection, improved coverage of difficult to sequence regions, and identification of pathogenic variants in non-coding regions and mitochondrial DNA. In addition to clinical impact, economic evaluation of a new technology is important before seeking scarce funding for its routine implementation into standard care. Here, we performed WGS in a WES-negative Mendelian cohort to determine the extent that WGS increases the diagnostic yield over WES and impacts diagnostic costs.

## Subjects and methods

### Cohort ascertainment

Individuals (*n* = 91; 64 families) with undiagnosed suspected Mendelian disorders were recruited from genetics units in New South Wales (NSW), Australia, from 2013 to 2017. Affected individuals had undergone a range of diagnostic investigations such as chromosomal microarray (CMA) in those with intellectual disability (ID), and in some, targeted gene sequencing, but no WES or WGS prior to this study [12]. Original WES studies were performed at the Kinghorn Centre for Clinical Genomics (KCCG) and the NSW Health Pathology Randwick Genomics Laboratory (RGL), with one family sequenced at Radboud University Medical Centre Nijmegen (RUMC). 41% of the original KCCG and RGL WES cohort had diagnostic findings [12, 13]. Following completion of WES analysis, individuals who remained undiagnosed were recruited for WGS, resulting in 38 families with 59 affected individuals and 41 unaffected first-degree relatives.

### Genomic sequencing and bioinformatics analysis

#### Original WES

WES was performed from 2013–17. RGL WES was performed on the Ion Proton using the Ion AmpliSeq Library kit V2 and PI Chip V2. KCCG WES was performed on the Illumina HiSeq 2500 [12]. Family 12 had WES at RUMC on the SOLiD platform as described previously [14]. Accredited WES bioinformatics pipelines were utilised including GAIA at RGL [13], in-house methods at the RUMC, and Seave at KCCG [12]. CNV analysis was performed using Conifer [15] or XHMM [16].

#### WGS

DNA was extracted from EDTA blood or cultured fibroblasts (2 families). Sequencing was performed on probands and unaffected relatives between 2016–17 on Illumina HiSeq X instruments on libraries generated using either the KAPA Hyper PCR-free kit (36 families) or the TruSeq Nano DNA kit (2 families). Variants were called after hs37d5 reference human genome [12] alignment using a BWA/GATK best practices pipeline. Single nucleotide variants (SNVs) and small insertion/deletion (indel) variants were annotated using VEP, converted into GEMINI databases [17], and loaded into the web-based variant filtration platform, Seave [18]. Sample gender and relatedness quality checks were performed using KING (v1.4) [19] and PLINK (v1.90b1g) [20].

Homozygosity mapping was performed using ROHmer (Puttick et al., manuscript in preparation). Mitochondrial SNV/indel analysis used mity [21] which runs FreeBayes (unpublished data). SVs including CNVs were identified using ClinSV [22], combining discordantly-mapping read pairs, split-mapping reads, and depth of coverage changes. SVs were annotated with population allele frequencies derived from 500 healthy controls [23], the 1,000 Genomes Project [24], and for protein-coding gene overlap.

### WGS variant prioritisation and interpretation

Nuclear SNVs and indels were filtered, prioritised, and interpreted by a clinical geneticist with genomic analysis expertise. Variants were discarded if the minor allele frequency was >2% (autosomal recessive (AR) or X-linked recessive inheritance) and >0.1% (autosomal dominant (AD)) in population databases, or with a predicted low impact on protein function. Candidate variant pathogenicity assessment was made using in silico prediction tools (SIFT [25], PolyPhen2 [26], PROVEAN [27], CADD [28]), and aggregate pathogenicity scores from Varcards [29]. Mitochondrial variants were filtered to known disease variants or overlapping phenotypes in MITOMAP [30]. SVs and CNVs were filtered by rarity, genotype-phenotype overlap, and family segregation.

Variants with genotype-phenotype correlation were reviewed for sequence quality in the Integrative Genomics Viewer (IGV) [31]. Candidate variants were classified by genetic pathologists utilising the American College of Medical Genetics (ACMG/AMP) guidelines and subsequently validated by Sanger sequencing, including family segregation, and reported if likely pathogenic/pathogenic [32].

### WES retrospective reanalysis

Retrospective WES reanalysis was performed on original WES data approximately 2 years following original WES analysis to determine if WGS diagnoses could be identified using contemporary techniques. If WGS-diagnosed variants were absent from WES reanalysis, an assessment was made of WES coverage over the critical region and the variant presence in VCF files.

### Health economic analysis

A health economic analysis was undertaken to understand the cost implications of genomic sequencing in Mendelian disorders. The incremental diagnostic and cost differences were analysed between the provision of WGS and WES for: (1) WES-negative individuals (38 families) and (2) individuals modelled as having had WES and WGS available with a contemporary analysis pipeline ab initio for the original 64 families (referred to as the *simulated* early genomic testing model).

1. Economic analysis for 38 WES-negative families. We calculated the incremental costs per additional WGS diagnosis when WES reanalysis was performed followed by WGS, and when only WGS was performed. As WES reanalysis on WES-negative cases is standard diagnostic care in the Australian healthcare system, WES reanalysis was the comparator for our analysis.
2. Economic analysis of the simulated early genomic testing model (64 families) Initial WES and WGS diagnostic rates were estimated using combined diagnoses from WES on the original cohort (64 families) and subsequently either contemporary WES reanalysis or WGS on the remaining 38 WES-negative families. We calculated the incremental costs per additional WGS diagnosis for contemporary WES followed by WGS and WGS alone, both compared to contemporary WES.

#### Sensitivity analysis

Diagnostic laboratory WES and WGS costs were sought in May 2020. For uniformity, laboratories offering WES and WGS were initially included (six laboratories), then refined to three laboratories offering singleton and trio studies for both technologies (Centogene, PerkinElmer, and Victorian Clinical Genetics Service (VCGS)). For primary analysis, VCGS laboratory costs were utilised for a local cost applicable for our base costs. WES and WGS costs were determined for varying family referral combinations such as singleton, trio, sibling pair, and multigenerational affected families. Costs for WES and WGS included sequencing, bioinformatics, interpretation, and reporting costs, and for WES reanalysis, bioinformatics, interpretation, and reporting costs. Additional affected individuals in families with multiple affected individuals incurred additional reporting costs. A sensitivity analysis was performed using the lowest and highest costs across the three laboratories (Supplementary Table 1). Only WES and WGS test costs were analysed for a direct comparison.

A bootstrapping method was used to assess the uncertainty of the economic model results for incremental costs and presented as 95% confidence intervals (CI). Replicated datasets were created by drawing a random sample of the WGS families 1000 times with replacement. Analyses were performed in Microsoft Excel and SAS version 9.4.

## Results

### Cohort demographics

Proband ages ranged from newborn to 73 years, with half of paediatric age, more affected males (64%), and parental consanguinity in 18%. Twenty families had a single affected proband, most undergoing trio sequencing (17/20). Eighteen families had multiple affected probands, with most (14/18) undergoing WGS of two affected family members. Patient demographics are summarised in Supplementary Table 2. The average time between original WES and WGS analysis was 1.8 years (SD ± 0.4) at KCCG, 2.4 years (SD ± 1.0) at RGL; combined 2.1 years (SD ± 0.7).

### WGS diagnoses were made in one-third of WES-negative families

WGS-based analysis diagnosed 34% of the previously undiagnosed cohort with one diagnosis per family (13/38 families; Table 1). Diagnoses were made in well-characterised diseases genes and due to SNVs and indels in 12 families and a CNV in 1 family. The greatest proportion of diagnoses by disease categories were haematological (2/2 families), skeletal (2/3 families), and ID (8/24 families; non-syndromic ID 3/7, syndromic ID 5/17) (Supplementary Fig. 1).

**Table 1:** WGS diagnoses, WES reanalysis diagnoses, and reasons why WES diagnoses missed.

F	Pt.	Phenotype		WGS diagnosis				Genomic testing		ES analysis/reanalysis		
		System	Description	Gene / Inheri-tance	Disease-causing variant	ACMG variant classification	Disorder	Original WES approach	WGS approach	Why missed on original WES	Retrospective contemporary WES reanalysis	WES coverage NovaSeq 6000
1	1,2	Haemat-ological	Oculocutanous albinism, absent platelet dense granules, ddx: Hermansky-Pudlak syndrome; parental consanguinity for both probands	*HPS6* AR, hom	NC_000010.11:g.102066504 G > T (GRCh38); NM_024747.5:c.1030 G > T; NP_079023.2:p.(Glu344Ter) SCV:001809937	Pathogenic: PVS1, PM2, PM3_supporting	Hermansky-Pudlak syndrome	Affected grandmother & grandson	Affected grandmother & grandson	Bioinformatics pipeline	Diagnosed. Updated bioinformatics pipeline.	
2	3,4,5	Haemat-ological	Macrothrombocytopaenia, platelet count 30-40 ×10^9^/L; abnormal platelet function & bleeding disorder. N/C	*ANKRD26* AD	NC_000010.11:g.27100442 G > A (GRCh38); NM_014915.2:c.-116C > T; exon 1 of 34 (5ʹUTR) position 57 of 414 SCV:001809938	Likely Pathogenic: RCV000851960.1	Thrombocytopenia 2 (MIM 188000)	3 affected individuals (cousins & uncle)	3 affected (cousins & uncle) & 1 unaffected (sibling)	5ʹUTR not covered	Undiagnosed. No coverage of 5ʹUTR	Genomic location covered
3	6,7,8	NS-ID	ID, epilepsy; consanguinity	*ALG11* AR, hom	NC_000013.11:g.52024914 T > C (GRCh38); NM_001004127.2:c.1184 T > C NP_001004127.2:p.(Met395Thr) SCV:001809939	Likely Pathogenic: PM1, PM2, PM3_supporting, PP3	Congenital disorder of glycosylation type 1p (MIM 613661)	Sibling pair	Affected sibling pair, aunt & unaffected parents	Reduced coverage	Undiagnosed. Reduced coverage.	Genomic location covered
4	9,10	NS-ID	ID; additional phenotype Dec 2017:Parkinson’s diagnosis, increased tremors, epilepsy, peripheral neuralgia, scoliosis?cerebral brain iron accumulation. N/C	*RAB39B* XLR	NC_000023.11:g.155264167_155265545del (GRCh38); Partial exon 1 deletion SCV:001809940	Pathogenic	X-linked mental retardation; Waisman syndrome (MIM: 300271; 311510)	Sibling pair	Sibling pair	CNV caller may have missed due to issues with exon 1 calling	Undiagnosed. Not identified through CNV analysis.	
5	11,12	NS-ID	Severe ID, absent speech, hypotonia microcephaly. N/C	*AP1S2* XLR	NC_000023.11:g.15854687 C > T (GRCh38); NM_001272071.2:c.-1 + 1 G > A p.(?)	Likely Pathogenic: PVS1, PM2	Mental retardation, X-linked syndromic 5 (MIM 304340)	Sibling pair	Sibling pair	Reduced coverage	Undiagnosed. Reduced coverage.	Reduced coverage of genomic location
6	13	S-ID	Severe ID, no speech, dysmorphic features: thick hair, epicanthic folds, mildly dysplastic helices, keratosis pilaris, dermatographia, pes planus, slender fingers & toes, flat nail beds, mild contractures of elbows & knees, mild obesity. N/C	*MSL3* AD *de novo*	NC_000023.11:g.11765731 T > A (GRCh38); chrX:g.11783850 T > A; NM_078629.3:c.1171 + 2 T > A SCV:001809941	Pathogenic: PVS1, PS2, PS3, PM2	X-linked neurodevelopmental delay, dysmorphism, and progressive neurological disorder	Trio	Trio	Bioinformatics pipeline	Diagnosed. Updated bioinformatics pipeline.	
7	14	S-ID	Moderate ID, adducted thumbs, hypotonia, abnormal aortic valve & prominent aortic root. N/C	*OGT* XLR	NC_000023.11:g.71537917 G > A (GRCh38); NM_181672.2:c.307 G > A; NP_858058.1:p.(Gly103Arg); In region with 2 prior pathogenic variants reported in N-terminal TPR domain SCV:001809942	Likely Pathogenic: PM1, PM2, PP2, PP3, PS4_supporting (RCV000624465.1)	X-linked mental retardation (MIM 300997)	Trio	Trio	Unknown disease gene association	Diagnosed. Interim disease gene publication.	
8	15,16	S-ID	ID, dysmorphic, epilepsy, osteoporosis and fractures (one proband). N/C	*SMS* XLR	NC_000023.11:g.21967298 A > G (GRCh38); NM_004595.4:c.152 A > G; NP_004586.2:p.(Tyr51Cys); Large Grantham 194 SCV:001809943	Likely pathogenic: PM2, PS4_moderate, PP4_moderate	Snyder-Robinson syndrome (MIM 309583)	Sibling pair	Sibling pair	Variant prioritization pipeline; stringent pathogenicity filter applied (CADD > / = 15)	Diagnosed. Updated variant prioritisation pipeline.	
9	17	S-ID	Severe ID, hypotonia, hypermobility, constipation, macrocephaly, prominent forehead, long face, slender hands & feet, prominent foetal finger pads, kyphoscoliosis, muscle wasting without significant weakness. N/C	*NFIX* AD *de novo*	NC_000019.10:g.13025339 C > T (GRCh38); NM_001271043.2:c.370 C > T; NP_001257972.1:p.(Arg124Trp) SCV:001809944	Pathogenic: PS2, PM1, PM2, PM5, PS4_moderate, PP2, PP3	Sotos type 2 (MIM 614753)	Trio	Trio	Bioinformatics pipeline; unlisted parental zygosity at genomic location	Diagnosed. Updated bioinformatics pipeline.	
10	18	S-ID	Marked polyhydramnios, umbilical cysts 20/40 USS, scalp oedema, severe microcephaly, bilateral coronal synostosis, eye proptosis, midface hypoplasia, cupid bow, cleft gum, lowset ears, bulbous digits, severe ascites, choanal stenosis, congenital pulmonary alveolar malformation type 3. N/C	*FGFR2* AD *de novo*	NC_000010.11:g.121515280 T > C (GRCh38); NM_000141.4:c.1124 A > G; NP_000132.3:p.(Tyr375Cys) SCV:001809945	Recurrent Pathogenic RCV000762799	Beare-Stevenson syndrome (extended *FGFR2* spectrum; MIM: 123790)	Trio	Trio	Bioinformatics pipeline	Undiagnosed. Variant calling failure in bioinformatics pipeline.	
11	19,20	Neurological	Neonatal deaths; microcephaly, cerebellar hypoplasia & simplified gyral pattern; oligohydramnios & pulmonary hypoplasia (pt.19); IUGR; Cardiac: globular heart & VSD (pt.19), cardiac hypertrophy (pt.20); CK 2,642 (pt.20); Eyes: posterior globe flattening, microphthalmia, optic atrophy, abnormal retinal vessels & fundi (pt.19), possible cataracts or vitreous anomaly (pt.20). N/C	*MIPEP* AR, comp het	(1) NC_000013.11:g.23837561 G > C (GRCh38); NM_005932.3:c.1534 C > G; NP_005923.2:p.(His512Asp); SCV:001809946 (2) NC_000013.11:g.23841336 A > G; NM_0059323:c.1259 T > C; NP_005923.2:p.(Leu420Pro) SCV:001809947	(1) Likely Pathogenic; PS4, PM1, PM3, PP3 (2) Likely pathogenic; PM1, PM2, PM3, PP3	Combined oxidative phosphorylation deficiency 31 (MIM 602241)	Sibling pair	Sibling pair	Unknown disease gene association; first publication within 1 week of analysis completion	Diagnosed. New disease gene publication.	
12	21	Skeletal	Sagittal craniosynostosis, ADHD. N/C	*ERF* AD	NC_000019.10:g.42254976 A > C (GRCh38); NM_005932.3:c.1259 T > C; Exon 1 donor canonical site	Pathogenic: PVS1, PM2, PP1_strong	Craniosynostosis (MIM: 600775)	Multiple affected individuals	Singleton	Reduced coverage	Undiagnosed. Reduced coverage.	Genomic location covered
13	22,23	Skeletal	Hernias, widened scars, fused radius/ulna, keratoconus, high myopia, subluxation of lenses, short stature; Weill-Marchesani syndrome? Parental consanguinity identified for pt. 22 after WGS.	*ASPH* 22: AR hom; 23: AR comp het	Patient 22: NC_000008.11:g.61548140 G > T (GRCh38); NM_004318.3:c.1695C > A; NP_004309.2:p.(Tyr565Ter); Patient 23: 1st variant and NC_000008.11:g.61526095 C > T (GRCh38); NM_004318.3:c.1782G > A; NP_004309.2:p.(Trp594Ter) SCV:001809948	(1) Pathogenic; PVS1, PM2, PM3_supporting (2) Pathogenic; PVS1, PM2, PM3	Traboulsi syndrome (MIM: 601552)	Multiple affected individuals	Multiple affected individuals	Variant prioritization pipeline; inheritance pattern assumption	Diagnosed. Analysis outside standard pipeline.	

*AD* autosomal dominant, *ADHD* attention deficit hyperactivity disorder, *AR* autosomal recessive, *CMA* chromosome microarray, *comp het* compound heterozygous, *F* family, *hom* homozygous, *L* litre, *N/C* non-consanguineous, *NS-ID* non-syndromic intellectual disability, *Pt.* patient, *S-ID* syndromic intellectual disability, *SCV* submitted record in ClinVar/accession number, *USS* ultrasound scan, *WES* whole exome sequencing, *WGS* whole genome sequencing, *XLR* X-linked recessive.

**Fig. 1:**
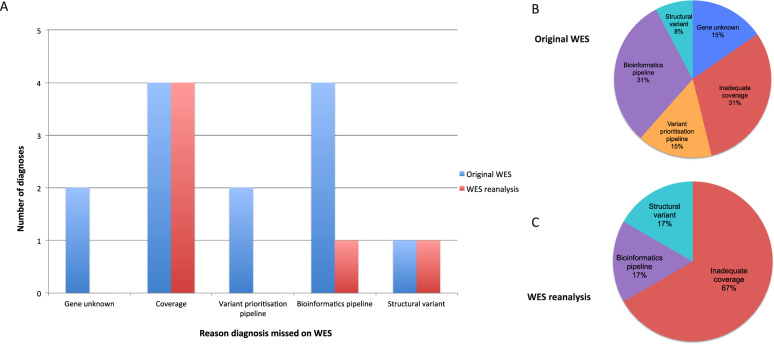
Explanations for WGS diagnoses missed by WES. **A** Bar chart comparing grouping of explanation for missed WES diagnoses made by WGS in blue for prior (original) WES and red for WES reanalysis. Visualisation of proportion of missed WES diagnoses based on reason for prior WES (**B**) and WES reanalysis (**C**).

WGS diagnoses were not detected through the original WES (Table 1, Fig. 1) due to a previously unknown gene-disease association (23%), insufficient sequencing coverage (31%), the variant prioritisation pipeline (15%), the bioinformatics pipeline (23%), or CNV detection (8%).

### WGS had increased sensitivity for detecting structural variation

**Fig. 2:**
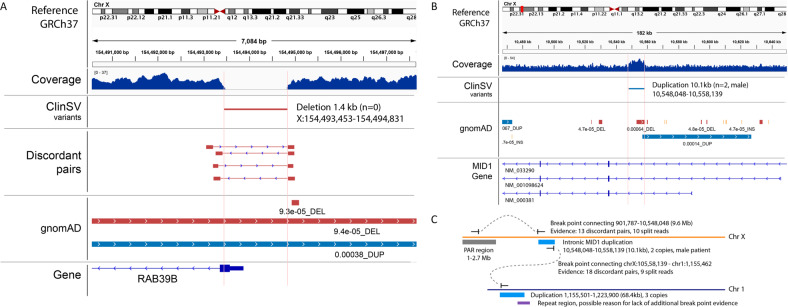
Structural variants identified through WGS. **A** *RAB39B* partial exon 1 deletion diagnosed through WGS. Simplified IGV (PMC3346182) screenshot showing ClinSV-detected deletion encompassing part of exon 1 and into the upstream untranslated region with supporting evidence. Tracks from top to bottom: sequencing read coverage, called structural variant, supporting discordant mapping read pairs, gnomAD variants with allele frequencies, gene models. There is no evidence of a similar deletion in gnomAD. Complex structural variant involving chromosome 1 and chromosome X in the region of *MID1:* (**B**) Simplified IGV (PMC3346182) screenshot showing intronic *MID1* duplication in an affected male; (**C**) Illustration of complex structural variant connecting parts of chromosome X and chromosome 1. The evidence suggests that a part of the pseudoautosomal regions (PAR) is connected with the intronic *MID1* duplication and a duplication on chromosome 1. The insertion point in the genome remains elusive, but is suspected to be on chromosome X due to evidence of X-linked inheritance in the family.

WGS data were evaluated to assess the impact of SVs on diagnostic yield. A 1.4 kb deletion encompassing part of exon 1 of *RAB39B* was identified in an X-linked ID family (Family 4, Table 1; Fig. 2A). The *RAB39B* deletion was validated in males using high-resolution CMA, adopting a lowered detection threshold of 4 probes from the standard 5 probes and a custom multiplex ligation-dependent probe amplification (MLPA). This deletion was missed on WES CNV analysis although visualisation of raw reads showed an absence of exon 1 coverage.

A family with Opitz G/BBB syndrome had a WGS-detected SV of uncertain significance. Prior *MID1* sequencing and WES were negative. There was evidence for two linked SV duplication events involving an intron of *MID1* on chromosome X and a region on chromosome 1 involving *SDF4* without a disease association. The X-linked pedigree is consistent with co-location of the duplications on chromosome X segregating with disease (Fig. 2B, C). Studies investigating the impact of the SV on *MID1* are in progress.

### Diagnoses made outside the standard variant analysis pipeline

Two diagnoses were made from bespoke analyses following initial negative routine variant prioritisation. Family 13 had a suspected X-linked or AD connective tissue disorder with features similar to Weill-Marchesani syndrome. Analysis for a shared candidate allele in an affected aunt and nephew was negative. Individual analyses were performed and, unexpectedly, a homozygous variant in *ASPH* (NM_004318.3:c.1695C > A; p.(Tyr565*)) associated with AR Traboulsi syndrome was identified in the aunt. This variant was present in the nephew in compound heterozygosity with a separate nonsense *ASPH* variant (NM_004318.3 c.1782G > A; p.(Trp594*)), demonstrating the presence of both homozygous and compound heterozygous *ASPH* alleles in the same family. Patient and pedigree review confirmed that their phenotype was consistent with Traboulsi syndrome, and that the aunt’s parents were third cousins. WES reanalysis confirmed that the family would have been diagnosed had this unusual mode of inheritance been considered.

Family 2 with AD macrothrombocytopaenia (Table 1) underwent an extended analysis to assess low impact variants in platelet disorder genes. This identified a previously reported pathogenic variant in the 5ʹUTR of *ANKRD26* (NM_014915.2:c.−116C > T) with a consistent haematological disease phenotype [33]. Sanger sequencing confirmed the variant segregated with disease. WES reanalysis did not identify this variant due to absent coverage of the 5ʹUTR in the earlier capture system. Improved coverage in a newer, alternate WES platform means this diagnosis would most likely have been made using current WES technology, provided variants of predicted low impact were prioritised through the pipeline (Supplementary Fig. 2).

### WES reanalysis would have detected over half of the WGS diagnoses

**Fig. 3:**
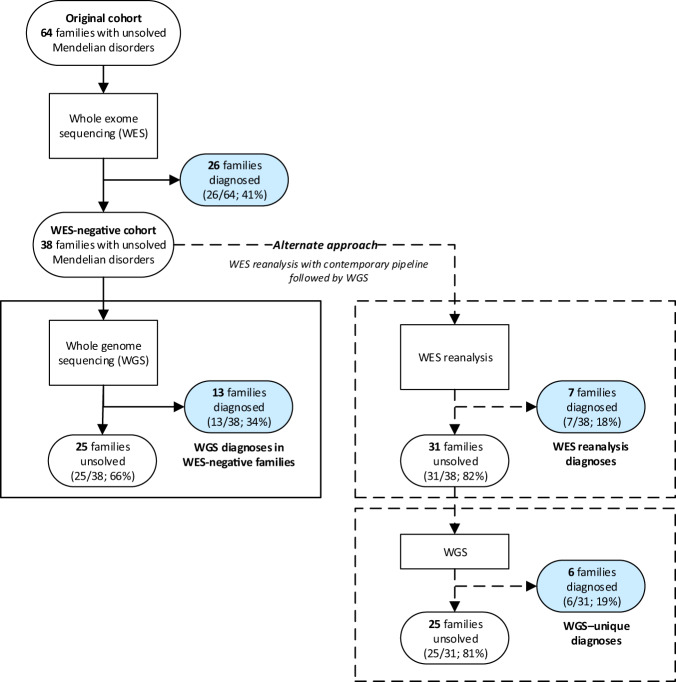
Comparison of diagnostic outcomes between WES, WES reanalysis with contemporary pipeline, and WGS. Blue shading represents families receiving a genomic diagnosis; grey shading represents undiagnosed families.

WES reanalysis identified the diagnosis in 7/13 families where WGS provided a diagnosis (54%; total WES-negative cohort 7/38 families, 18%; Table 1; Fig. 3). Therefore, WGS provided an additional diagnostic yield following WES reanalysis of 19% of WES-negative families (6/31 remaining families). The majority of new WES diagnoses were due to improvements in the bioinformatics/variant filtering pipeline (4/7; Table 1: families 1, 6, 8, 9), followed by new disease gene identification (2/7; families 7, 11), and analysis outside the standard pipeline (1/7; family 13). In those that remained undiagnosed after WES reanalysis (6/13 families), insufficient coverage of the diagnostic variant was the main cause (4/6; Table 1: families 2, 3, 5, 12), and one missed CNV (family 4) and one bioinformatics pipeline error (family 10).

### WGS versus WES as an initial genomic test

We estimated the diagnostic yield of WGS over a contemporary WES pipeline in the original cohort of 64 genomic-naïve families (Fig. 3). To do this, original WES diagnoses and additional diagnosed families from WGS or contemporary WES reanalysis were combined, assuming all original WES diagnoses would have been made with contemporary WES and WGS. From this analysis, a contemporary WES pipeline would have yielded a 52% diagnostic yield (33/64 families: 26 original WES families and 7 additional from contemporary WES pipeline). Had WGS been performed at the outset, 61% of families would have been diagnosed (39/64 families; 26 original WES, 13 additional WGS), resulting in an additional 9% yield over contemporary WES, unique to WGS.

### Health economic analysis

#### Economic analysis for WES-negative families

The incremental cost per additional WGS diagnosis following WES reanalysis was AU$36,710 (£19,407; US$23,727) (95% CI: AU$20,607; $112,902) compared to WES reanalysis alone. For WGS alone, the incremental cost per additional diagnosis was AU$41,916 (£22,159; US$27,093) (95% CI: AU$22,790; $128,107) compared to WES reanalysis alone. This pathway conferred the greatest costs with the same diagnostic rate as the WES reanalysis followed by WGS pathway.

#### Economic analysis for the *simulated* early genomic testing model

*WGS following initial WES* (Table 2, ii): for each additional (incremental) WGS diagnosis over initial WES, the cost would be AU$36,710 (£19,407; US$23,727) (95% CI: AU$20,946; $112,942). *Initial WGS* (Table 2, iii): for each incremental initial WGS diagnosis, there would be an additional cost of AU$29,708 (£15,705; US$19,201) (95% CI: AU$16,612; $90,195) compared to initial WES. Thus, of the two WGS options, WGS as the initial test was the best value for money, producing the same diagnoses at a lower cost than WGS following WES.

**Table 2:** Economic comparative analysis of WGS with WES and WES reanalysis.

	Unresolved Mendelian families from original WES analysis (*n* = 38 families)			WGS versus WES as initial genomic test (*n* = 64 families; includes 38 WES-negative families)		
	Contemporary WES reanalysis on WES-negative cases (a)	Contemporary WES reanalysis followed by WGS^a^ (b)	WGS on WES-negative cases (c)	WES (contemporary) (i)	WES followed by WGS (ii)	WGS^b^ (iii)
Diagnoses with WES reanalysis	7	7 (7/38; 18%)	—	—	—	—
Diagnoses with contemporary WES	—	—	—	33	33 (33/64; 52%)	—
Diagnoses with WGS	—	6 (6/31; 19%)	13	—	6 (6/31; 19%)	39
Total diagnosed	7 (7/38; 18%)	13 (13/38; 34%)	13 (13/38; 34%)	33 (33/64; 52%)	39 (39/64; 61%)	39 (39/64; 61%)
Total cost of WES reanalysis	AU$15,727 (£8,314; US$10,164)	AU$15,727 (£8,314; US$10,164)	—	—	—	—
Total cost of contemporary WES	—	—	—	AU$252,945 (£133,720; US$163,482)	AU$252,945 (£133,720; US$163,482)	—
Total cost of WGS	—	AU$220,268 (£116,445; US$142,362)	AU$267,240 (£141,277; US$172,722)	—	AU$220,268 (£116,445; US$142,362)	AU$431,191 (£227,950; US$278,685)
Total cost	AU$15,727 (£8,314; US$10,164)	AU$235,993 (£124,758; US$152,526)	AU$267,240 (£141,277; US$172,722)	AU$252,945 (£133,720; US$163,482)	AU$473,211 (£250,164; US$305,844)	AU$431,191 (£227,950; US$278,685)
Incremental cost per additional WGS diagnosis						
- Compared to WES reanalysis	Reference	AU$36,710 (£19,407; US$23,727)	AU$41916 (£22,159; US$27,093)	—	—	—
(95% confidence interval)		(AU$20,607; 112,902)	(AU$22,790; 128,107)			
- Compared to initial WES	—	—	—	Reference	AU$36,710 (£19,407; US$23727)	AU$29,708 (£15,705; US$19,201)
(95% confidence interval)					(AU$20,946; 112,942)	(AU$16,612; 90,195)

38 WES-negative unsolved Mendelian families are presented on the left, showing comparative costs for three alternative approaches to further genomic analysis: (a) contemporary WES reanalysis, (b) contemporary WES reanalysis followed by WGS, (c) WGS only. Original 64 unsolved Mendelian families are presented on the right with a simulated early genomic testing model. Comparative costs are shown for three initial genomic approaches: (i) WES (contemporary), (ii) WES followed by WGS, (iii) WGS only. The number of diagnosed families from each technology are shown as well as the total costs and the incremental cost for each additional WGS diagnosis. Costs are in Australian Dollars (laboratory costs obtained as of 13th May 2020 and conversion to British Pounds, and US Dollars with a rate of 1 AUD = 0.53 GBP/0.65 USD/0.60 EUR (https://www.xe.com/currencytables/?from=AUD&date=2020-05-13#table-section)).

^a^Includes cost of WES reanalysis and no WGS cost for families diagnosed through ES reanalysis.

^b^It was assumed that all cases diagnosed with WES would have been diagnosed with WGS.

#### Sensitivity analysis

There were substantial differences in genomic sequencing costs between laboratories, with more widely divergent WGS costs. Trio WGS costs ranged from AU$7,557 (£3,995; US$4,884) (VCGS laboratory) to AU$11,446 (£6,051; US$7,398) (Centogene laboratory), and trio WES ranged from AU$3,713 (£1,963; US$2,400) (PerkinElmer laboratory) to AU$4,345 (£2,297; US$2,808) (Centogene laboratory). Accordingly, we undertook a sensitivity analysis to determine what impacts the use of higher or lower WES and WGS costs may have on the results.

##### Economic analysis for WES-negative families

The incremental cost per additional WGS diagnosis following WES reanalysis ranged from AU$36,659 (£19,380; US$23,693) (lowest WGS cost) to AU$53,307 (£28,181; US$34,453) (highest WGS cost) when compared to WES reanalysis alone, with the cost of WES reanalysis unchanged. The sensitivity analysis showed that the conclusions for WES-negative families would not be altered by lower or higher genomic costs.

##### Economic analysis for the simulated early genomic testing model

One-way sensitivity analysis of the incremental cost for each additional initial WGS diagnosis compared to initial WES was performed for a range of available WES and WGS costs (Supplementary Fig. 3). The result is more sensitive to WGS costs, with the incremental cost ranging from AU$28,262 (£14,941; US$18,266) to AU$60,681 (£32,079; US$39,219) per additional WGS diagnosis, primarily driven by the wider range of commercially available costs for WGS compared to WES.

## Discussion

In this Mendelian disorder cohort, WGS resulted in a diagnosis in one third (34%; 13/38 families) of undiagnosed families who had previously had WES. However, when controlling for factors such as improvements to gene-disease knowledge and genomic pipelines through contemporary WES reanalysis, the WGS diagnostic yield reduced to 19% (6/31 remaining families). If WGS was applied as an initial test to our original cohort of 64 undiagnosed Mendelian families, the increased diagnostic yield unique to WGS was 9% relative to contemporary WES. The majority of the WGS diagnostic gains (4/6 diagnoses; Fig. 1C) were due to reduced WES coverage of critical regions that may be solved through an improved WES platform. Inspection of sequencing coverage in unrelated individuals using the newer Illumina NovaSeq 6000 ES Agilent CREv2 showed adequate coverage for variant identification in 3 of the 4 missed WES diagnoses (Supplementary Fig. 4). Although there was a low detection rate of pathogenic SVs in this study, this may increase with time as more clinically important SVs are characterised and thus influence WGS diagnostic yields over WES [34, 35].

### Solving the undiagnosed

Understanding why genomic diagnoses are missed can lead to alterations to genomic pipelines and improved Mendelian disorder diagnosis. A WGS diagnosis in a deceased foetus with suspected Raine syndrome followed multiple sequential non-informative investigations including prenatal CMA, *FAM20C* sequencing and MLPA, a craniosynostosis panel, and WES. On WGS, a de novo pathogenic *FGFR2* variant (p.Y375C) was identified, diagnosing Beare-Stevenson syndrome, conferring a greatly reduced reproductive recurrence risk compared to the suspected AR disorder. The craniosynostosis panel had included *FGFR2*, but not the critical exon, and the missed WES diagnosis was due to a failure of the variant caller despite good sequencing coverage, which has been subsequently addressed.

Generic genomic filtering pipelines may rely on assumptions about inheritance patterns or predicted protein impacts. Failure to identify a molecular aetiology after a familial analysis should prompt consideration of an alternative analytical method, such as singleton proband analysis in the family with Traboulsi syndrome. Similarly, incorporating known Mendelian disorder disease-causing variants from ClinVar that are bioinformatically predicted to be of low impact, improves variant detection. Accessing specialist gene-disease knowledge will be important for recognition of such variation.

WES reanalysis remains valuable in increasing diagnostic yields in unsolved cases, with an additional diagnostic rate of 11% (7 of 64 families) made over an approximate 2-year period [12]. However, reanalysis of WES obtained from older platforms may be ineffective in some unsolved individuals due to overall reduced sequencing coverage compared to contemporary platforms. There remains a diagnostic gap with WES for smaller SVs that is best approached through non-WES methodologies such as exon-level arrays or WGS. While contemporary WES coverage has improved, including slightly expanded coverage of non-coding regions containing pathogenic variation [5, 6, 36], WGS enables the unbiased detection of non-coding variants without the limitation of target enrichment based on potentially outdated gene annotations. Although less is understood how non-coding region variation impacts biological function, there are numerous examples of deep intronic variation affecting gene splicing [7] and other pathogenic non-coding variants [5, 6] such as the 5ʹUTR *ANKRD26* variant in this study. Proof of causation for novel non-coding variation is challenging but higher throughput methodologies for functional studies may lower costs and improve understanding of such variation, making diagnostic reporting more feasible and increasing the importance of WGS [37]. While we have compared current diagnostic WES and WGS pipelines, there are a number of techniques such as improved splicing prediction tools [38] and RNAseq [36] that are not yet routinely available but have potential to further increase diagnostic rates over current WES and WGS.

### WES or WGS as an initial genomic diagnostic test?

Variants assessed for disease diagnosis are almost exclusively in coding regions and so it has been argued that a well performed contemporary WES study is a cost-effective screen and the best first-line methodology [12, 39]. However, we may be moving towards a time when WGS will be adopted as a first-line test [40]. The main limitation of WES is a lower sensitivity for detecting structural variation, particularly complex variation [39]. Further, when considering the maximum diagnostic yield alone, this study and others have shown that WGS boosts the diagnostic yield in WES-negative Mendelian disorder cohorts [8–11]. The magnitude of this diagnostic increase depends on the modernity of the WES approach relating to exome enrichment, analytic pipelines, and the likelihood of CNVs or the presence of an unusual genomic mechanism. There is evidence that small CNVs may be more important in Mendelian disease diagnosis than previously recognised [35] so the increased sensitivity of WGS for CNV detection is advantageous. The combination of WES with newer technological platforms such as long-read sequencing could result in an increased diagnostic sensitivity for CNVs without the higher costs of performing WGS.

Decisions about when to use WES and WGS remain important because there is a trade-off between the lower cost of WES and the higher diagnostic yield of WGS. To date, there have been few studies on comparing the relative costs of WGS with WES or after WES reanalysis [41]. The economic analyses in this study show that the economic decision whether to use WES or WGS in part depends upon whether prior genomic testing has occurred. If additional diagnoses are sought when WES has been performed previously, the *lowest cost* use of resources is to perform WES reanalysis. However, to achieve *maximal* diagnoses, the most cost-effective strategy is to perform WGS after WES reanalysis, with an incremental cost per additional WGS diagnosis of AU$36,710 (£19,407; US$23,727) in this study. This strategy incurs a lower cost than performing WGS after original WES without WES reanalysis, with the same diagnostic yield.

For people who have not had genomic testing, the most cost-effective strategy for *maximal* diagnoses is to perform initial WGS, with an incremental cost of AU$29,708 (£15,705; US$19,201) per WGS diagnosis. However, acknowledging that some diagnoses will be missed and that not all jurisdictions have access to the required resources for WGS, the *lowest cost* pathways are to perform WES reanalysis in WES-negative individuals and initial WES in people who have not had genomic testing. It is important to note that the cost differentials between WES and WGS may be specific to this study cohort and that there is no universally acknowledged willingness-to-pay-threshold for a diagnosis [42]. Further, the additional expenditure for each WGS diagnosis achieved may still result in downstream health and social cost savings, which, over a lifetime, may dwarf the costs of WGS [43].

The implications of diagnoses for families on quality of life outcomes, management change, access to reproductive technologies, eligibility for services, access to support groups and the impact on both health and social costs all need to be considered when allocating scarce resources. The economic analysis in this study lacks information about such outcomes that would provide information on quality adjusted life years (QALYs) and allow for a cost utility analysis. Further, we have not calculated the costs of additional investigations that may be incurred following a negative WES result compared to WGS. However, the economic analysis does provide important information about the financial resource implications of implementing WES and WGS, when considering those test costs alone.

In addition to balancing test cost and maximising diagnoses, the clinical scenario also influences genomic test choice. In settings where there is a high chance of intervention if a genomic diagnosis is made, it can be argued that WGS, with the maximal chance of diagnosis, should be chosen. Such scenarios may include the acutely unwell children in the neonatal or paediatric intensive care units (NICU/PICU) [44], or for urgent reproductive situations such as an at-risk pregnancy. However, such decisions are not made in isolation, with availability and resourcing impacting the option to provide, or choice of genomic testing, even in urgent clinical scenarios.

WGS is the optimal genomic test choice to maximise the diagnostic rate in Mendelian disorders across all clinical scenarios. However, accepting a small reduction in diagnostic yield, WES with reanalysis confers the lowest costs. Whether WES or WGS is utilised will depend on the clinical scenario and local resourcing and availability.

## Supplementary information

Supplementary Material

## Data Availability

Web-based variant filtration platform, Seave, software is available (https://github.com/KCCG/seave). Causative variants have been deposited to the ClinVar database (SUB10158418).
