# Supplementary material for: Whole exome and genome sequencing in mendelian disorders: a diagnostic and health economic analysis
Source: Eur J Hum Genet. 2022 Aug 15;30(10):1121–31. doi: 10.1038/s41431-022-01162-2 (PMC9553973; doi:10.1038/s41431-022-01162-2)
Supplement: Supplementary file 1 — Supplementary Material [file 41431_2022_1162_MOESM1_ESM.pdf]

Whole Exome and Genome Sequencing in Mendelian Disorders: A Diagnostic and Health Economic Analysis

Supplementary Material

Supplementary Figure 1. WGS families in cohort by body system and diagnosis status.

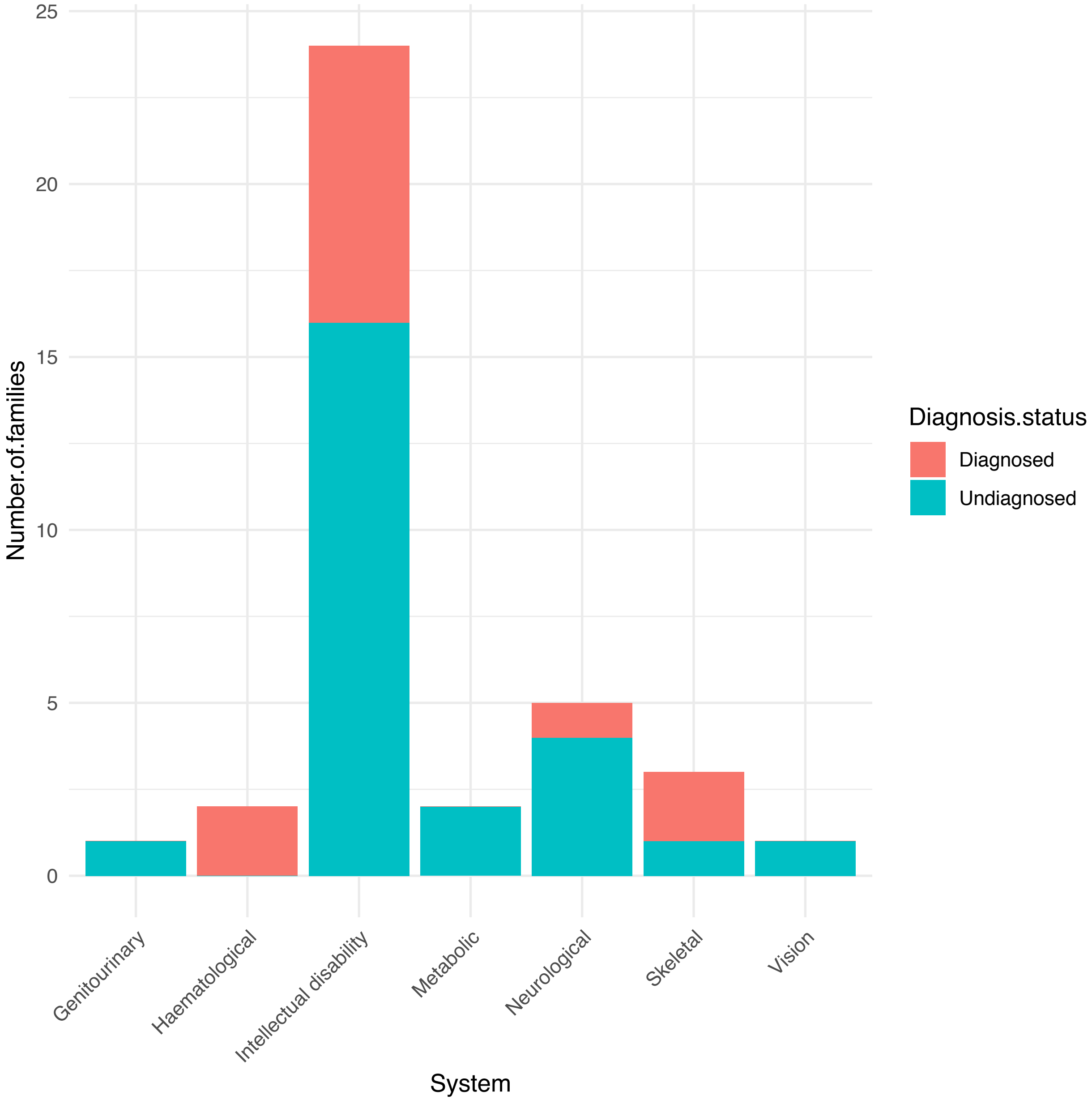

Supplementary Figure 2. Variation in platform coverage of 5'UTR of *ANKRD26*. (A) Integrative genomics viewer (IGV) screenshot of WGS Illumina data in 3 affected individuals with thrombocytopenia in one family; green vertical line represents heterozygous 5'UTR pathogenic variant identified. (B) Ion Proton WES coverage in the 3 affected family members of same region showing an absence of aligned data across 5'UTR. (C) Unrelated individuals using Illumina WES platform with capture across *ANKRD26* 5'UTR. (D) WES data in same region from unrelated individuals using a recent Ion Proton platform showing absence of data.

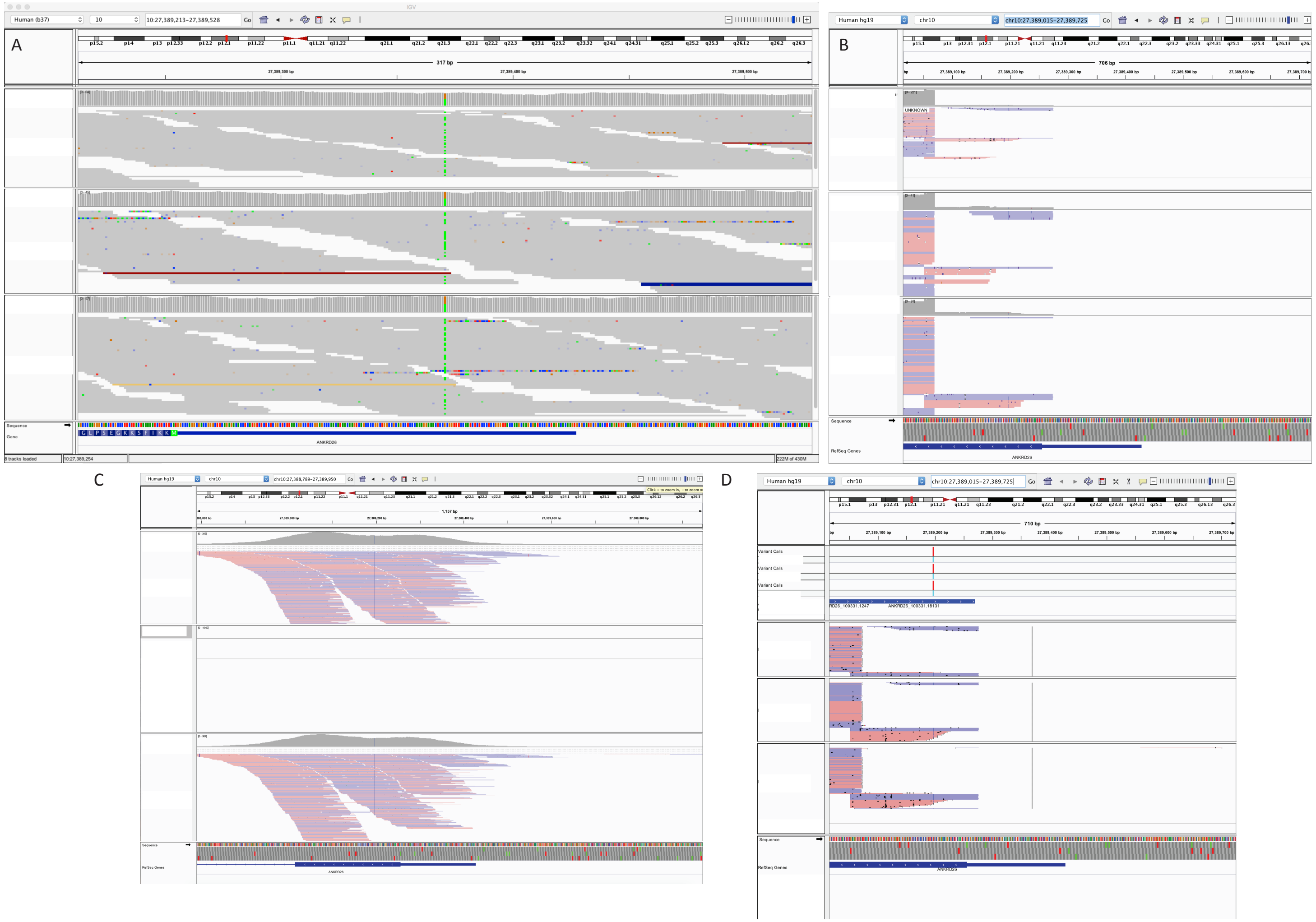

Supplementary Figure 3. Incremental costs of additional initial genomic diagnoses are more sensitive to WGS costs. Tornado plot showing a one-way sensitivity economic analysis in simulated early genomic testing cohort. Incremental costs (AU$) for each additional initial WGS diagnosis are compared to initial WES for a range of WES and WGS costs from the available laboratories. The increased sensitivity to WGS costs are primarily driven by the wider range of WGS costs.

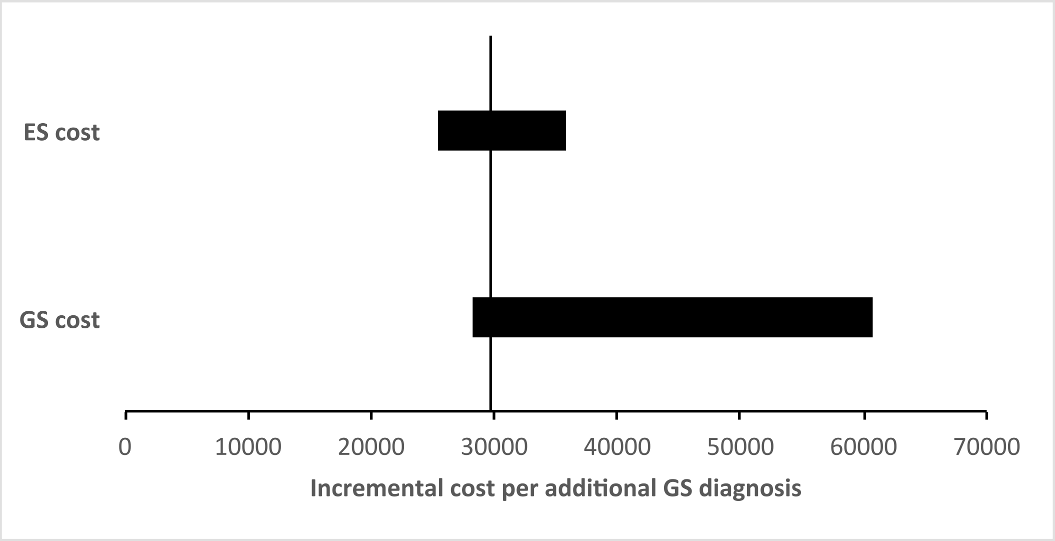

Supplementary Figure 4. Contemporary WES coverage of genomic locations of WGS diagnoses missed on older WES. (A) *ANKRD26:* chr10(GRCh37):g.27389371G>A (chr10(GRCh38):g.27100442); Family 2 (B) *ALG11*: chr13(GRCh37):g.52599050T>C (chr13(GRCh38):g.52024914); Family 3 (C) *AP1S2*: chrX(GRCh37):g.15872810C>T (chrX(GRCh38):g.15854687); Family 5 (D) *ERF*: chr19(GRCh37):g.42759128A>C (chr19(GRCh38):g.42254976); Family 12. Sequencing performed in unrelated individuals using Illumina NovaSeq ES CREv2.

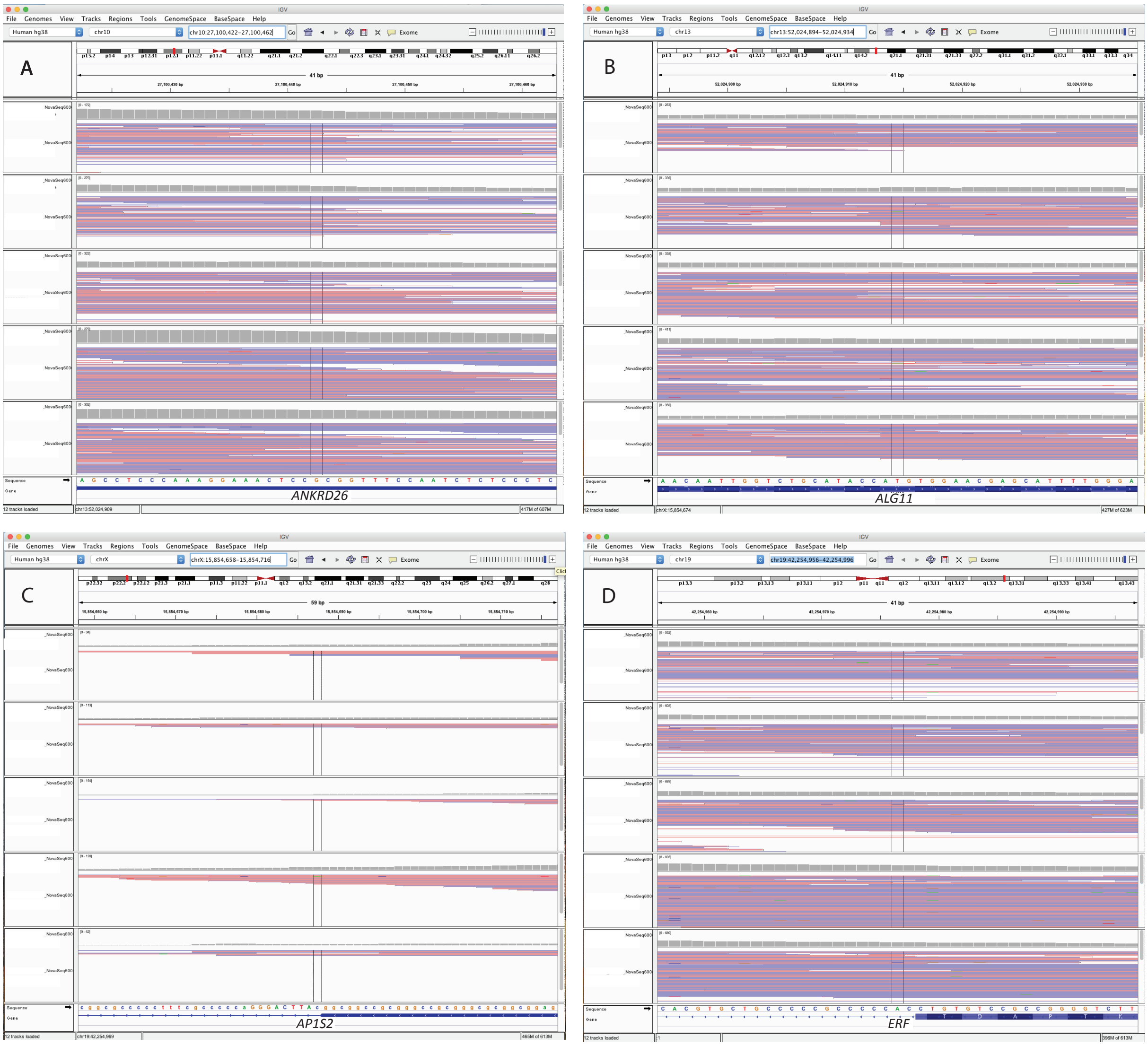

Supplementary Table 1. Test costs utilised for economic analyses. 6 diagnostic laboratories offering WES & WGS were identified and contacted by email for pricing structure. Of these, 3 laboratories offered both singleton WES and WGS (Centogene; Perkin Elmer; VCGS). Prices were converted to Australian Dollars as of 13th May 2020 (https://www.xe.com/currencytables/?from=AUD&date=2020-05-13#table-section). Costs were broken-down into various family structures consistent with at least 1 referred family in the Mendelian cohorts. Average costs between laboratories were calculated. Costs from Victorian Clinical Genetics Service (VCGS), an Australian laboratory, were selected as the local diagnostic laboratory offering both WES and WGS.

Test	Cost breakdown by: family structure / sequencing / analysis / report	VCGS (Australian lab; AU Dollars)	Costs for sensitivity analysis	
			Lowest	Highest
WES	Singleton	3166	1838	3166
	2 probands	3778	3258	3778
	3 probands	4391	4391	5570
	Trio	4187	3713	4846
	Trio + 1 proband	4800	4800	6016
	Trio + 2 probands	5412	5412	7427
	Trio (2 probands, 1 unaffected)	4289	4289	4846
	3 probands + 1 unaffected	4902	4902	6016
	Additional sequencing / individual	511	/	/
	Additional proband (sequencing + report)	613	/	/
WES reanalysis	Per report	102	102	309
	Per analysis	357	309	357
	E.g. 2 probands	460	460	619
WGS	Singleton	4391	3868	4929
	2 probands	6076	6076	8271
	3 probands	7761	7761	11604
	Trio	7557	7557	11446
	Trio + 1 proband	9242	9242	14705
	Trio + 2 probands	10927	10927	14705
	Trio (2 probands, 1 unaffected)	7659	7659	11446
	3 probands + 1 unaffected	9446	9446	14705
	Additional sequencing / individual	1583	/	/
	Additional proband (sequencing + report)	1685	/	/

Supplementary Table 2. Comparison of cohort demographics between WES-negative and 64 family genomic-naïve cohort.

Key: a, As at January 1 2017; b, less than 16 years; NS-ID, non-syndromic intellectual disability; S-ID, syndromic intellectual disability

		WES-negative cohort	Genomic-naïve cohort (Includes 38 WES-negative families)
Families		38	64
Probands		59	91
Proband Sex	Male	64% (38/59)	62% (56/91)
	Female	36% (21/59)	38% (35/91)
Proband average age (years)[a]		22	19
Proportion pediatric age range[a,b]		49% (29/59)	57% (52/91)
Family referral structure for sequencing	Trio study	55% (21/38)	52% (33/64)
	Singleton	8% (3/38)	16% (10/64)
	Multiple affected individuals	37% (14/38)	33% (21/64)
Mendelian disorder grouping	Genitourinary	3% (1/38)	2% (1/64)
	Hematological	5% (2/38)	6% (4/64)
	Immunological	0%	3% (2/64)
	Metabolic	5% (2/38)	5% (3/64)
	Neurological	13% (5/38)	14% (9/64)
	NS-ID	18% (7/38)	13% (8/64)
	S-ID	45% (17/38)	44% (28/64)
	Skeletal	8% (3/38)	9% (6/64)
	Syndromic	0%	2% (1/64)
	Visual	3% (1/38)	3% (2/64)
